# A majority of uninfected adults show preexisting antibody reactivity against SARS-CoV-2

**DOI:** 10.1172/jci.insight.146316

**Published:** 2021-04-22

**Authors:** Abdelilah Majdoubi, Christina Michalski, Sarah E. O’Connell, Sarah Dada, Sandeep Narpala, Jean Gelinas, Disha Mehta, Claire Cheung, Dirk F.H. Winkler, Manjula Basappa, Aaron C. Liu, Matthias Görges, Vilte E. Barakauskas, Mike Irvine, Jennifer Mehalko, Dominic Esposito, Inna Sekirov, Agatha N. Jassem, David M. Goldfarb, Steven Pelech, Daniel C. Douek, Adrian B. McDermott, Pascal M. Lavoie

**Affiliations:** 1 BC Children’s Hospital Research Institute, Vancouver, British Columbia, Canada.; 2 Department of Pediatrics, University of British Columbia, Vancouver, British Columbia, Canada.; 3 Vaccine Research Center, National Institute of Allergy and Infectious Diseases, NIH, Bethesda, Maryland, USA.; 4 Department of Anesthesiology, Surrey Memorial Hospital (SMH), Surrey, British Columbia, Canada.; 5 Department of Anesthesiology & Pain Medicine, University of Alberta, Edmonton, Alberta, Canada.; 6 Department of Anesthesiology, Pharmacology and Therapeutics, University of British Columbia, Vancouver, British Columbia, Canada.; 7 Kinexus Bioinformatics Corporation, Vancouver, British Columbia, Canada.; 8 Vaccine Evaluation Centre, BC Children’s Hospital Research Institute, Vancouver, British Columbia.; 9 Department of Pathology and Laboratory Medicine, University of British Columbia, Vancouver, British Columbia, Canada.; 10 National Cancer Institute RAS Initiative, Cancer Research Technology Program, Frederick National Laboratory for Cancer Research, Leidos Biomedical Research Inc., Frederick, Maryland, USA.; 11 British Columbia Centre for Disease Control (CDC) Public Health Laboratory, Vancouver, British Columbia, Canada.; 12 Division of Medical Microbiology, Department of Pathology and Laboratory Medicine, and; 13 Department of Medicine, University of British Columbia, Vancouver, Canada.

**Keywords:** COVID-19, Immunology, Adaptive immunity

## Abstract

Preexisting cross-reactivity to SARS-CoV-2 occurs in the absence of prior viral exposure. However, this has been difficult to quantify at the population level due to a lack of reliably defined seroreactivity thresholds. Using an orthogonal antibody testing approach, we estimated that about 0.6% of nontriaged adults from the greater Vancouver, Canada, area between May 17 and June 19, 2020, showed clear evidence of a prior SARS-CoV-2 infection, after adjusting for false-positive and false-negative test results. Using a highly sensitive multiplex assay and positive/negative thresholds established in infants in whom maternal antibodies have waned, we determined that more than 90% of uninfected adults showed antibody reactivity against the spike protein, receptor-binding domain (RBD), N-terminal domain (NTD), or the nucleocapsid (N) protein from SARS-CoV-2. This seroreactivity was evenly distributed across age and sex, correlated with circulating coronaviruses’ reactivity, and was partially outcompeted by soluble circulating coronaviruses’ spike. Using a custom SARS-CoV-2 peptide mapping array, we found that this antibody reactivity broadly mapped to spike and to conserved nonstructural viral proteins. We conclude that most adults display preexisting antibody cross-reactivity against SARS-CoV-2, which further supports investigation of how this may impact the clinical severity of COVID-19 or SARS-CoV-2 vaccine responses.

## Graphical abstract

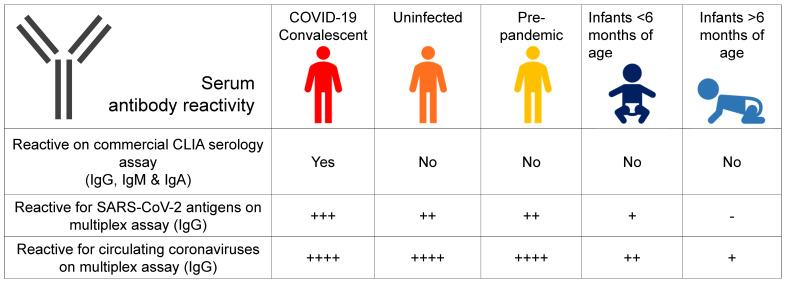

## Introduction

Coronavirus disease 2019 (COVID-19) was declared a global pandemic on March 11, 2020, and has resulted in almost 100 million confirmed cases and 2.1 million deaths worldwide as of January 24, 2021. Almost all individuals infected with SARS-CoV-2 seroconvert within 2–3 weeks, with the spike and nucleocapsid (N) proteins eliciting the strongest responses (1, 2). While much attention has focused on defining immune reactivity in individuals after infection, other data indicate that many individuals show preexisting SARS-CoV-2 cross-reactive T and B cells without prior exposure to the virus (3–5). However, the extent of preexisting SARS-CoV-2 antibody reactivity at the population level has been difficult to estimate, due to a lack of assay sensitivity (6) and clearly definable background thresholds to identify meaningful seroreactivity among individuals who have been unexposed to the virus (7).

There are 4 circulating coronaviruses predating COVID-19 that cause up to 30% of seasonal upper respiratory tract infections (8). The spike proteins of β-coronaviruses HKU1 and OC43 exhibit approximately 40% sequence similarity, whereas the α-coronaviruses NL63 and 229E exhibit approximately 30% structural similarity with SARS-CoV-2 (9). The common occurrence of circulating coronaviruses year after year and their structural similarity with SARS-CoV-2 raises the possibility that the former may stimulate cross-reactive responses toward SARS-CoV-2 and that this heterotopic immunity may impact clinical susceptibility to COVID-19 and/or modulate responses to the SARS-CoV-2 vaccine (10, 11).

The main objective of this study was to estimate the extent of the preexisting seroreactivity against SARS-CoV-2 in the general adult population and its relationship to circulating coronaviruses. To confirm that SARS-CoV-2 antibody reactivity in uninfected adults was genuinely cross-reactive and not due to widespread unreported, asymptomatic SARS-CoV-2 circulation, we similarly assayed sera collected prior to the emergence of SARS-CoV-2 and from infants before and after maternal antibodies have waned. In addition, we used a SPOT peptide array to map this antibody reactivity on the SARS-CoV-2 proteome.

## Results

### Study population.

In total, 276 healthy adults were recruited for this cohort between May 17 and June 19, 2020. The demographic characteristics and geographical area of residence of participants are shown in Supplemental Table 1 (supplemental material available online with this article; https://doi.org/10.1172/jci.insight.146316DS1) and Supplemental Figure 1, respectively. The majority (*n* = 196; 71%) were health care workers. Less than half had traveled outside of British Columbia (BC) since January 1,2020, to the USA, Europe, Iran, the Caribbean, Australia, Mexico and Japan. Two individuals had a history of PCR-confirmed COVID-19.

### Prevalence of prior SARS-CoV-2 infection in the study population.

**Figure 1:**
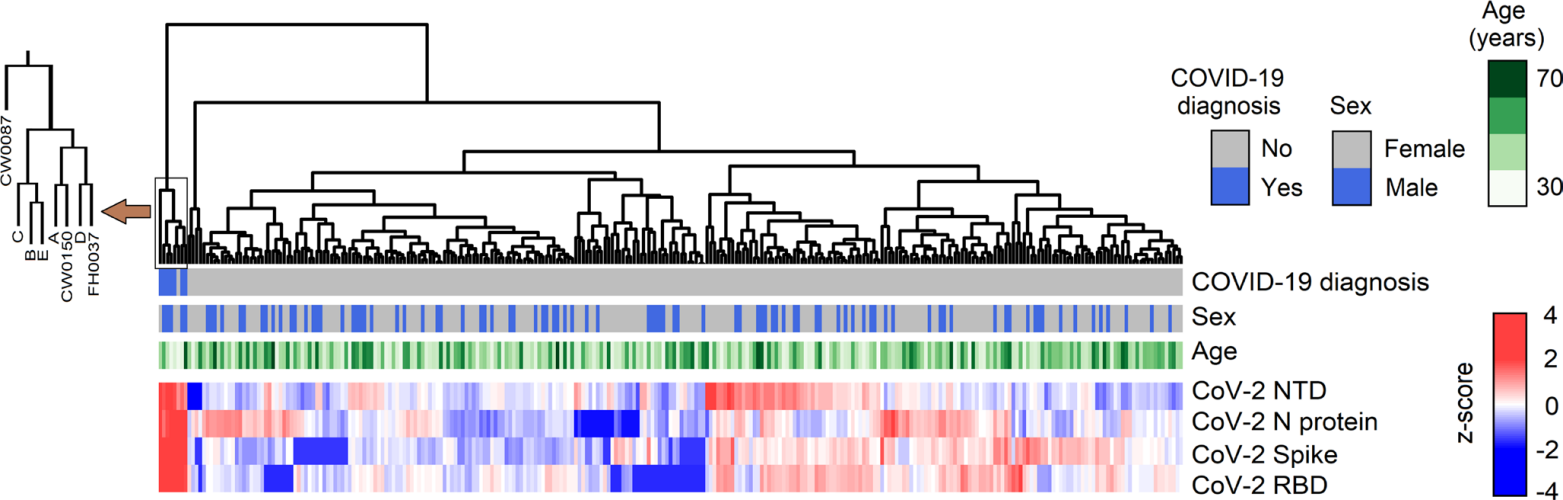
Hierarchical clustering of individual based on serum SARS-CoV-2 antibody reactivity profiles. COVID-19 diagnosis identifies convalescing individuals who had a positive viral test by PCR. This figure combines data from 276 study participants plus the 5 COVID-19 convalescent control sera. Color scale represents antibody reactivity as a *Z* score.

To estimate the proportion of individuals who had been previously infected with SARS-CoV-2, we used a multiplex assay to profile antibody reactivity against 4 viral antigens: the whole SARS-CoV-2 spike protein, its N-terminal domain (NTD) and receptor-binding domain (RBD), and the N protein. Clustering analysis based on antibody reactivity for these 4 antigens identified that 3 individuals (CW087, CW0150, FH0037) and 5 control sera from convalescent COVID-19 patients (controls A, B, C, D and E) clustered together, separately from the rest of the cohort (Figure 1). The antibody reactivity profile of these 8 distinct sera showed high reactivity against all 4 SARS-CoV-2 antigens, whereas all other individuals showed variable antibody reactivity against either spike, RBD, or the N protein (Supplemental Figure 2).

The 3 individuals (CW087, CW0150, FH0037) who clustered with the 5 control sera included the 2 individuals who had a history of PCR-confirmed COVID-19, plus an asymptomatic woman who was not aware she had COVID-19 initially but later identified that she had been in contact with a COVID-19 case about 90 days prior to serology testing for this study (Supplemental Table 2).

All sera from the cohort who displayed above-the-mean antibody reactivity for at least 1 of the 4 SARS-CoV-2 antigens (i.e., for a total of 222 out of 276 individuals) were further tested with a commercial diagnostic commercial chemiluminescent (CLIA) assay, which recognizes the spike protein S1 antigen (Supplemental Figure 3). With this assay, the same 3 individuals (CW087, CW0150, FH0037), plus the 5 control sera mentioned above, tested positive. Therefore, based upon these data, it appeared that 3 of 276 participants (1.1%) showed clear evidence of a previous infection with SARS-CoV-2. After adjusting for bias by using point estimates of specificity and sensitivity of the CLIA assay, we estimated that the prevalence of a previous SARS-CoV-2 infection was 0.60% (95%CI, 0%–2.71%) in this cohort.

### Antibody reactivity to circulating coronaviruses.

The multiplex assay also included quantification of antibody reactivity against the spike proteins of circulating coronaviruses (OC43, HKU1, NL63, 229E). All individuals showed high antibody reactivity against the spike proteins from these circulating coronaviruses (Supplemental Figure 2). We used correlation analyses to understand the relationship between the antibody reactivity against SARS-CoV-2 and circulating coronaviruses. Among the 273 seronegative individuals, we detected significant correlations between antibody reactivity to SARS-CoV-2 spike, as well as spike proteins from HKU1, NL63, and 229E, but not OC43 (Supplemental Table 3 and Supplemental Figure 4).

### Specificity of SARS-CoV-2 antibody reactivity in uninfected individuals.

**Figure 2:**
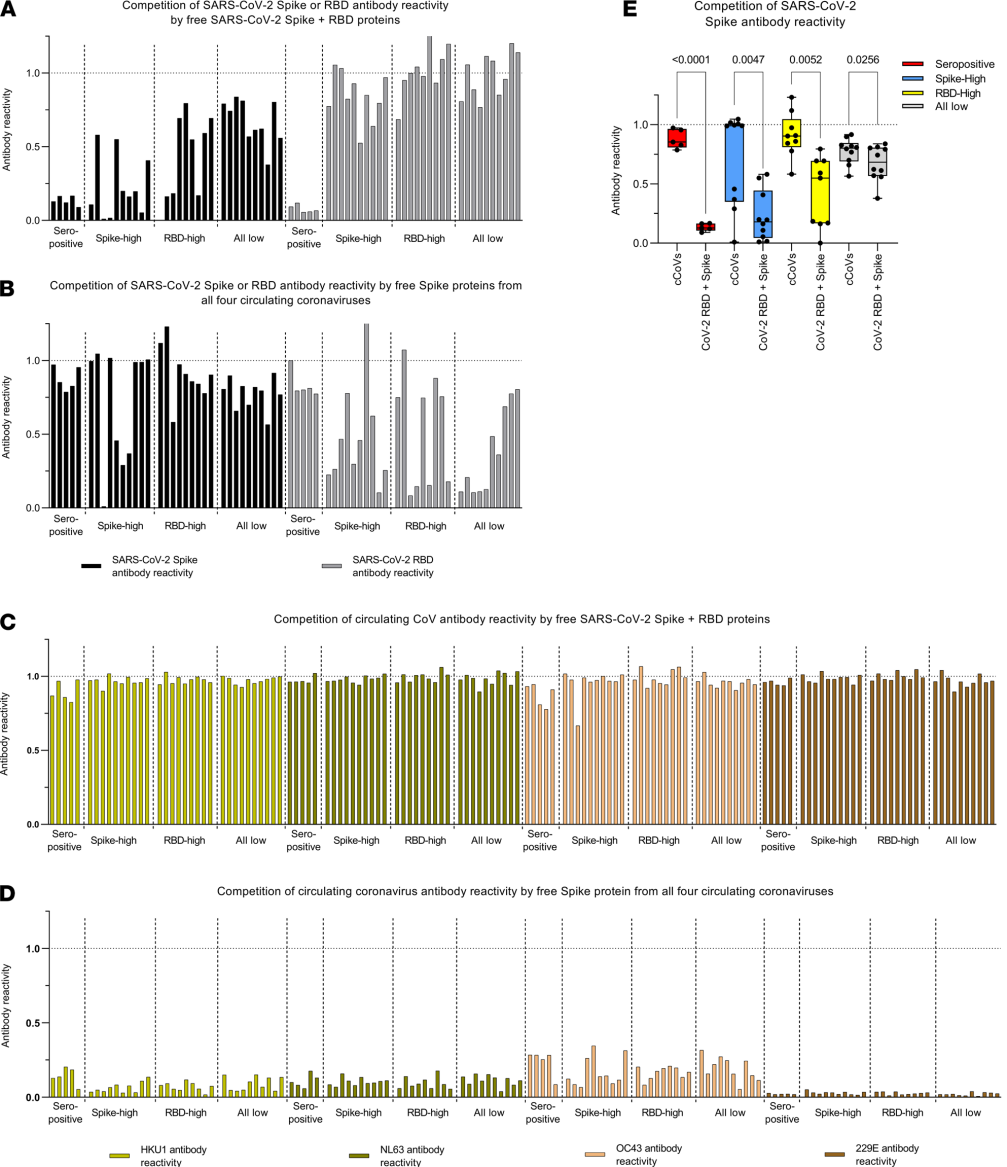
Specificity of SARS-CoV-2 antibody reactivity. (**A** and **B**) Competition of SARS-CoV-2 spike and RBD antibody reactivity by SARS-CoV-2 spike and RBD proteins (**A**) or by circulating coronaviruses (cCoVs) spike proteins (**B**). (**C** and **D**) Competition of cCoVs spike antibody reactivity by SARS-CoV-2 spike and RBD proteins (**C**) or cCoVs spike proteins (**D**). (**E**) Competition of SARS-CoV-2 spike antibody reactivity by SARS-CoV-2 spike and RBD proteins or cCoVs spike proteins, in COVID-19 convalescent sera (seropositive, *n* = 5), or sera from uninfected individuals who showed highest SARS-CoV-2 spike (*n* = 10) and high RBD (*n* = 9), or lowest SARS-CoV-2 spike and RBD antibody reactivity (all low; *n* = 10). All values represent the ratios of antibody reactivity in competed samples over the antibody reactivity measured in absence of competing proteins (dash line). One sample in the RBD-high group failed, and these data are not shown. In **E**, data are represented as boxes (25th to 75th percentile, line at median) and whiskers (minimum to maximum); comparisons were made using 2-tailed paired *t* tests.

Next, we conducted competition experiments to exclude the possibility that the antibody reactivity against SARS-CoV-2 in uninfected individuals was due to nonspecific binding in the multiplex assay and to assess whether this antibody reactivity may represent cross-reactive antibody responses to circulating coronaviruses. To these ends, we determined whether the antibody reactivity against antigens in the multiplex assay could be outcompeted using either a cocktail of free SARS-CoV-2 RBD and full-length spike proteins — or the spike proteins from all 4 other circulating coronavirus spike proteins (OC43, HKU1, NL63, and 229E) pooled (Figure 2). Antibody reactivity was measured on serial dilutions from selected COVID-19 convalescent and uninfected sera selected on the basis of a high reactivity to full-length SARS-CoV-2 spike protein, its RBD, or its low reactivity to both of these antigens. As expected, the high SARS-CoV-2 spike and RBD antibody reactivity in COVID-19 convalescent sera was efficiently outcompeted by free SARS-CoV-2 spike and RBD proteins, but not by other free circulating coronavirus spike proteins (Figure 2, A and B).

Moreover, antibody reactivity to SARS-CoV-2 spike and RBD was partially outcompeted by circulating coronavirus spikes in uninfected individuals — this latter finding supports the argument that at least some of this SARS-CoV-2 antibody reactivity represented cross-reactivity toward circulating coronaviruses. Conversely, reactivity to circulating coronavirus spike proteins was efficiently outcompeted by spike protein from circulating coronaviruses but not by SARS-CoV-2 spike and RBD proteins (Figure 2, C and D). Therefore, this experiment confirms that SARS-CoV-2 spike and RBD antibody reactivity in uninfected individuals is saturatable, essentially excluding nonspecific binding in the multiplex assay. Interestingly, competition of SARS-CoV-2 spike antibody reactivity was higher in uninfected individuals with higher detectable SARS-CoV-2 spike or RBD antibody reactivity compared with individuals who showed low reactivity against these 2 antigens (Figure 2E). Unexpectedly, antibody reactivity against SARS-CoV-2 RBD in uninfected individuals was not efficiently competed by a fixed amount of SARS-CoV-2 RBD.

### Seroreactivity thresholds defined in sera from immunologically naive infants.

**Figure 3:**
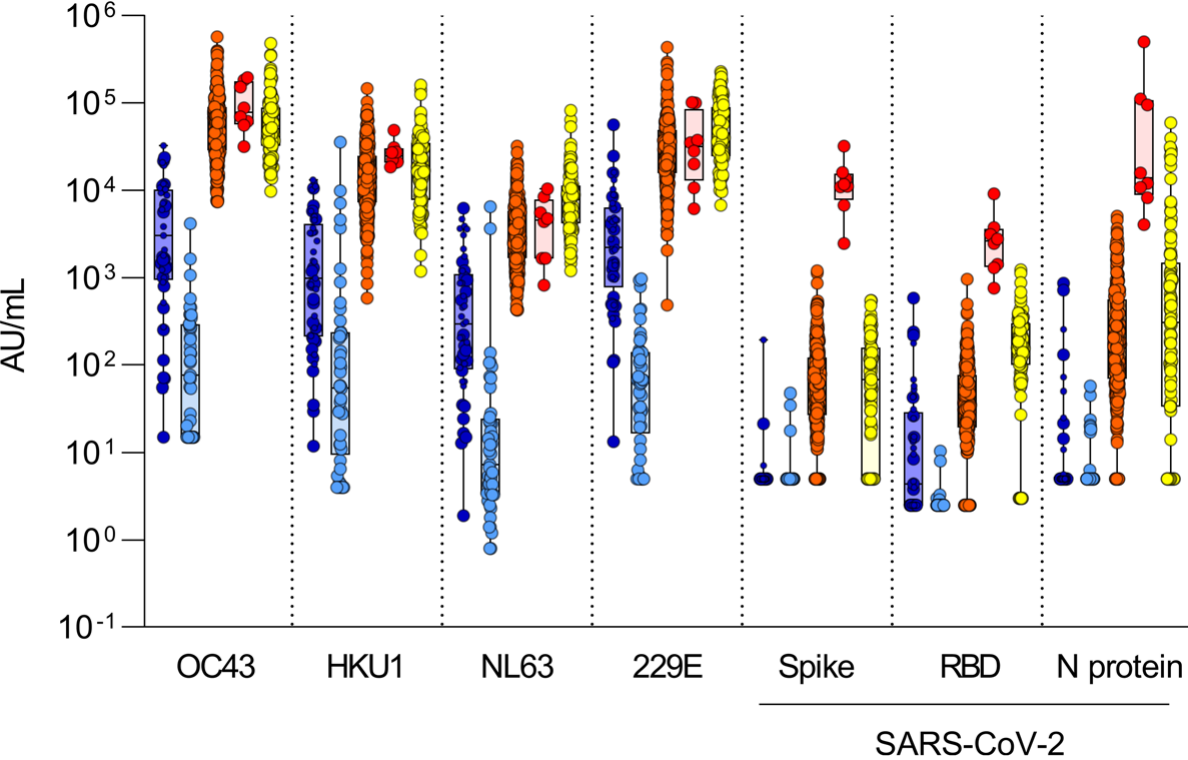
Thresholds of antibody reactivity based on infants’ sera. Comparison of antibody reactivity (AU/mL) in infants sampled before 6 months of age (darker blue) and again about 8 months later (lighter blue; *n* = 45), in SARS-CoV-2–uninfected (orange; *n* = 273), in SARS-CoV-2–infected (convalescent) adults (red; *n* = 8), and in prepandemic sera (yellow; *n* = 99). Infants sampled before the pandemic (January 1, 2020) are represented by the larger circle symbols, whereas infants sampled after January 1, 2020, are shown using the small circle symbols. Boxes represent median with 25th and 75th percentiles with positive/negative antibody reactivity thresholds for SARS-CoV-2 spike calculated at the 99th percentile for value distribution (10.00 AU/mL), RBD (10.00 AU/mL), and N protein (10.00 AU/mL) as 10^(mean^ ^log[antibody^ ^reactivity]^^+^^SD^ ^log^ ^[antibody^ ^reactivity] × 2.33)^ in infants’ sera. Antibody detection for NTD was low and inconsistent between experiments; therefore, the data are not presented and reactivity thresholds were not calculated.

To unequivocally distinguish uninfected individuals who could have SARS-CoV-2 antibody reactivity, we defined the background of antibody reactivity of sera in the multiplex assay. We reasoned that infants would be immunologically naive, with the exception of maternal antibodies that are expected to wane gradually after birth; thus, their sera can be used to define antibody reactivity thresholds in uninfected adults in the multiplex assay. Using this assay, we measured antibody reactivity of sera from 45 infants less than 6 months of age and repeated this measurement in the same infants approximately 8 months later, after BC’s lockdown period (Figure 3); the study included 21 infants in whom the first sera were obtained before the pandemic (i.e., before January 2020). In infant sera, reactivity to circulating coronaviruses was uniformly detected in the first set of blood samples, albeit at much lower levels than adults. In the second infant sample sets taken after July 1, 2020, antibody recognition of circulating coronaviruses had decreased to approximately 1000-fold lower levels compared with adults, consistent with a waning of maternal antibodies (Supplemental Figure 5). When comparing antibody reactivity of SARS-CoV-2 in the second postnatal infant sera, levels were up to 100-fold higher in uninfected adults compared with infants, regarding the different SARS-CoV-2 antigens (Figure 3).

Thus, these second infant samples allowed us to define effective thresholds for SARS-CoV-2 antibody reactivity in uninfected adults (Figure 3). Based on infants’ sera, we estimate that between 90% and 99% of adults show positive antibody reactivity for SARS-CoV-2 spike, RBD, or the N antigen. Prepandemic sera showed similar antibody reactivity, therefore excluding the possibility that the reactivity in adults after the first pandemic wave is due to undiagnosed exposures to the virus in the study population. This baseline, preexisting SARS-CoV-2 cross-reactivity in uninfected adults was evenly distributed according to age, sex, travel history, or whether participants were healthcare workers (HCW), and the data were independent of participants’ reporting “COVID-19-like” symptoms (Supplemental Figure 6).

### Further characterization of SARS-CoV-2 antibody reactivity in uninfected adults.

**Figure 4:**
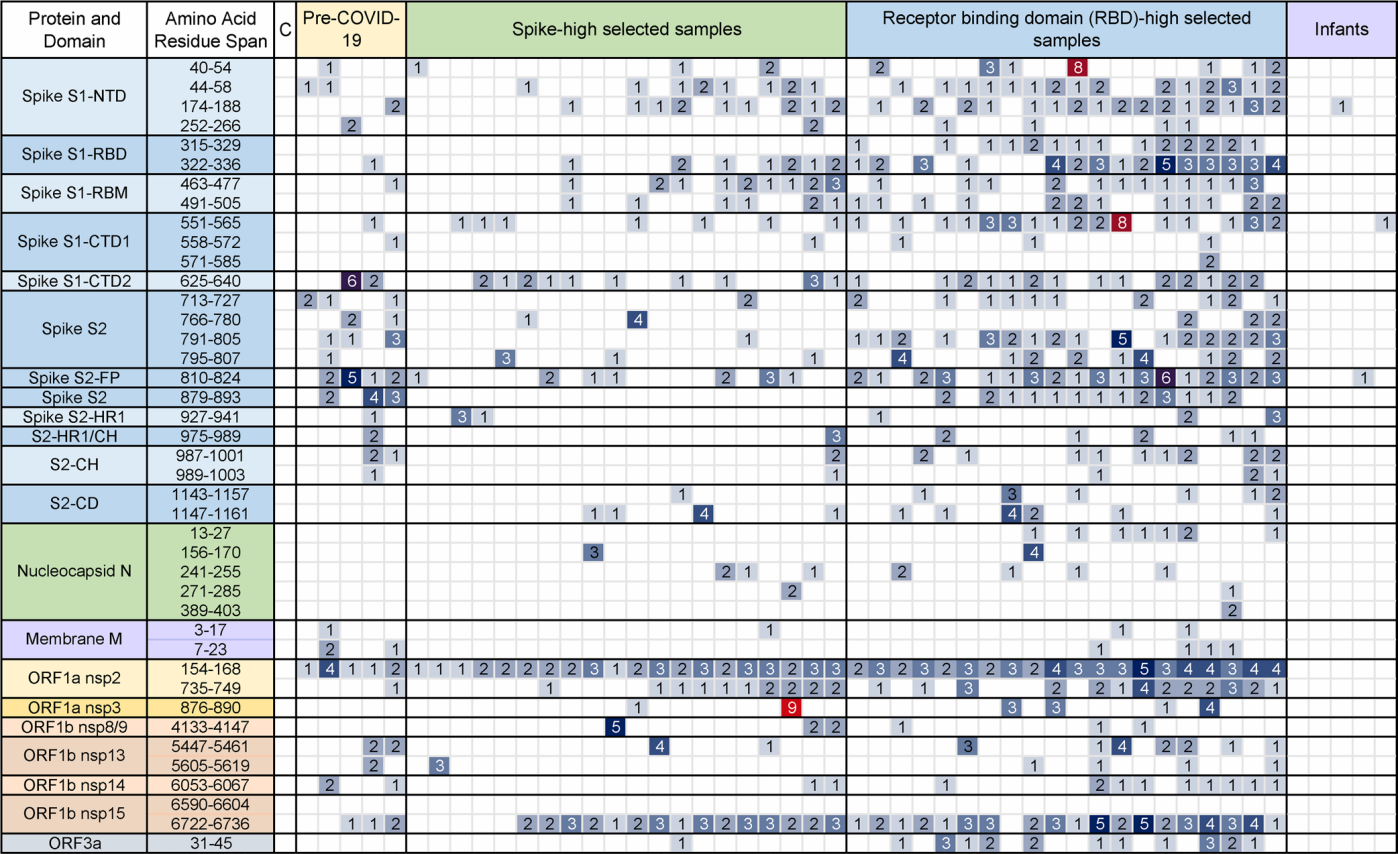
Mapping of SARS-CoV-2 antibody reactivity in sera from uninfected individuals. Serum antibody binding to 15-mer peptides distributed across the SARS-CoV-2 proteome or an IgG-binding peptide (positive control), from 5 randomly selected prepandemic samples, adults showing high level of spike or RBD reactivity (*n* = 20 each), or infants (*n* = 5). Values represent signals on a scale from 0 to 10, after subtracting background. The column labeled C shows the immunoreactivity signal in the absence of sera, but with the addition of anti–human IgA, IgM, and IgG horse-radish peroxidase–coupled secondary antibody. In the spike protein, labels indicate the N-terminal (NTD), C-terminal (CTD), or receptor-binding (RBD) domains, receptor-binding motif (RBM), heptad repeat sequence (HR1), central helix (CH), or connector domain (CD); N, nucleocapsid protein; M, membrane protein; ORF, open-reading frame polypeptide proteins; nsp, nonstructural proteins.

To map this antibody reactivity on the viral proteome, we used a SPOT array assay where peptides broadly covering the SARS-CoV-2 proteome were directly synthesized on a cellulose membrane (Supplemental Figure 7). To enrich for high-affinity antibodies, sera from individuals who showed high spike or RBD antibody reactivity were compared with infant samples. As shown in Figure 4, we detected high antibody reactivity against nonstructural proteins, particularly the nonstructural protein 2 (nsp2) and nsp15 encoded in the replicase polypeptides ORF1a and ORF1b. RBD-high samples showed the strongest antibody reactivity encompassing RBD, as well as the S1 and the S2 peptides, indicating a diverse anti–SARS-CoV-2 antibody reactivity linked to a high RBD antibody cross-reactivity. This cross-reactivity was also detected in randomly selected prepandemic sera, which demonstrated preexisting recognition prior to the SARS-CoV-2 pandemic. Importantly, we detected no antibody reactivity against any viral peptides in infants’ sera.

## Discussion

In this study, we estimated that 0.60% (95%CI, 0%–2.71%) of the study population showed clear evidence of a prior infection with SARS-CoV-2. The combination of a highly specific commercial CLIA assay and a highly sensitive multiplex assay allowed us to distinguish individuals who have been infected with SARS-CoV-2 from those who have not. This prevalence of SARS-CoV-2 infections was identical to the 0.55% prevalence reported by the BC CDC on 885 residual sera obtained from an outpatient laboratory network in the Lower Mainland of BC between May 15 and May 27, 2020. Data from the BC CDC represent a wider geographical catchment and do not specifically target HCW (12). The current study confirms that COVID-19 transmission in BC after the first wave was low, even among HCW, contrasting with a high seroprevalence reported among HCW in other studies (13–15), which may be attributed to the very low number of total tested cases in BC during the first wave.

The main finding in this study is that, at a population level, the vast majority of adults show antibody reactivity against SARS-CoV-2 antigens. BC reported its first COVID-19 case on January 29, 2020, with the first documented case of community transmission on March 5, 2020. The first pandemic wave peaked between the third week of March and late April 2020 (11). As of May 17, only 2445 diagnosed COVID-19 cases (approximately 49 of 100,000 population) had been reported in BC after the first wave, which was the lowest rate in Canada and one of the lowest rates in North America. Because of a relatively low number of COVID-19 cases in BC after the first wave, it is extremely unlikely that this antibody reactivity results from a direct exposure to SARS-CoV-2. Moreover, findings of similar antibody reactivity in prepandemic adult sera and from sera obtained from infants younger than 1 year of age confirms that we are detecting genuine cross-reactivity rather than reactivity to SARS-CoV-2 from asymptomatic COVID-19 cases.

Our findings are consistent with another study in which prepandemic sera exhibited cross-reactive IgG antibody reactivity with conserved epitopes in SARS-CoV-2 proteins (S2 and N) (5). The higher prevalence of preexisting antibody reactivity in uninfected adults in our cohort compared with this previous study may be explained by the high sensitivity of our assay and evidence of positive seroreactivity in those individuals informed by the infant sera. However, whereas these previous studies have quantified cross-reactivity in selected sera, to the best of our knowledge, the current study is the first to determine SARS-CoV-2 antibody reactivity at the population level. The fact that we measured antibody reactivity between infected and uninfected individuals in the same population and time period in the current study also eliminates recruitment or sampling biases and is another major strength of this study.

The presence of preexisting SARS-CoV-2 antibody reactivity in uninfected individuals in the current study is consistent with the detection of T cell reactivity against SARS-CoV-2 in about 40% of uninfected individuals (3, 4). This raises an important question: what is the antigenic source of this antibody reactivity? Competition experiments and correlatives analyses indicate that it may, in part, be attributable to cross-reactivity against circulating coronaviruses. Most humans become infected with circulating coronaviruses by their second year of age (16). On the one hand, correlations between SARS-CoV-2 and antibody reactivity against either HKU1, N63L, or 229E, but not OC43, could reflect seasonal variations in recent exposure to common coronaviruses (10, 17). On the other hand, the high antibody reactivity to SARS-CoV in individuals in this study likely represents cross-reactivity due to the higher (>75%) sequence similarity between SARS-CoV and SARS-CoV-2 (18, 19), rather than a previous exposure to SARS-CoV.

The data presented in this study shed light on another important question: what region of the virus does this preexisting antibody reactivity bind to? We found in our peptide mapping experiments that it is broadly distributed across the viral proteome, including whole spike, and proteins encoding the viral replication complex. The binding to ORF polypeptides could be a sign of infection by circulating coronaviruses that share conserved sequences with SARS-CoV-2. High antibody reactivity against nonstructural ORF proteins was reported in another study using a VirScan peptide mapping approach on prepandemic sera (6). However, due to a lower sensitivity of the assay, antibody reactivity against spike was not detected in the latter study. Here, we confirm that this preexisting antibody reactivity involves structural external elements of the virus in both epitope mapping and competition experiments.

It is unclear whether this antibody reactivity may confer clinical benefits — for instance, modulating the severity of a SARS-CoV-2 infection. Data indicate that a past circulating coronavirus infection may decrease the severity of a subsequent SARS-CoV-2 infection (20). Others have linked preexisting seroreactivity against circulating coronaviruses to increased SARS-CoV-2 pseudovirus neutralization in vitro (5), although this remains debated. Individuals with high RBD reactivity showed the most structurally diverse antibody reactivity against spike epitopes, which may enhance viral clearance in addition to the improved neutralizing activity specific to RBD-specific antibodies. Indeed, strong antibody response to RBD have been linked to improved clinical outcomes from COVID-19 (21). However, reactivity against RBD in the multiplex assay was not competed by soluble RBD in uninfected individuals, despite that this reactivity was almost completely abrogated in COVID-19 convalescent sera. The latter finding is consistent with another study that showed that preexisting antibody reactivity against SARS-CoV-2 in prepandemic sera could be efficiently competed by a soluble S1 (that contains the RBD domain) but not a soluble S2 subunit of the spike protein (5). Notably, we were also unable to detect ACE2 receptor binding inhibition from sera of uninfected individuals (not shown), which could indicate that the preexisting antibody reactivity against SARS-CoV-2 in uninfected adults represents an excess of low-affinity antibodies that have poor overall viral neutralizing potential. This may not be surprising given that viral neutralization improves generally with affinity maturation, an antigen-driven process that requires cognate interaction by B cells, in collaboration with follicular T cells. Similarly, preexisting, highly variable low-avidivity SARS-CoV-2 CD4 memory T cells cross-reactive to circulating coronaviruses appeared less protective in uninfected adults (22). More studies are needed to understand the origin of preexisting SARS-CoV-2 antibodies and their impact on COVID-19 severity.

In conclusion, this study reveals common preexisting, broadly reactive SARS-CoV-2 antibodies in uninfected adults. These findings warrant larger studies to understand how these antibodies affect the severity of COVID-19, as well as the quality and longevity of responses to SARS-CoV-2 vaccines.

## Methods

### Study design.

Prospective cross-sectional study after the first pandemic wave in BC.

### Participants.

Adults over 18 years of age from the greater Vancouver metropolitan area were included if they did not have active COVID-19, did not require self-isolation as per BC provincial public measures, or had recovered from COVID-19 at least 14 days prior to the study visit and blood collection. Blood was drawn in gold-top serum separator tubes with polymer gel (BD Biosciences, catalog 367989); after at least 30 minutes of clotting at room temperature, the blood sample was then centrifuged at 1400*g* for 10 minutes at room temperature to obtain serum aliquots that were frozen at –80°C within 4 hours of collection. Adult prepandemic sera were all obtained before January 1, 2020. Infants’ sera were collected before discharge from hospital at birth (first sample) and after June 11, 2020 (second sample), as part of a study examining antibody responses to respiratory viruses.

### Recruitment.

Greater Vancouver is the main urban center in BC and the third largest metropolitan area in Canada, with a population of 2.5 million. Study participants were invited by an email sent to clinical departments of the BC Children’s & Women’s (C&W) Hospitals (the largest pediatric referral center in BC, located in Vancouver, and where no cases of COVID-19 were admitted during the pandemic’s first wave) and its affiliated BC Children’s Research Institute (BCCHR). The study was also advertised to hospitalists, anesthesiologists, and critical care physicians at SMH (located approximately 27 km from Vancouver). To minimize recruitment bias, all adults who responded to the invitation email and returned their signed consent form were enrolled sequentially and invited to give a blood sample, without triaging. Blood samples were collected between May 17 and June 19, 2020.

### Study size.

Since there were little population seroprevalence data available at the time and none in BC or Canada, no a priori sample size calculation was performed. The recruitment period was, therefore, defined by convenience over a 3-week period of enrollment, in order to obtain baseline data.

### Multiplex antibody assay.

A highly sensitive multiplex (10-plex) assay (Meso Scale Diagnostics, catalog K15369U) where each antigen is “spotted” into a single well of a 96-well plate (23) was used to measure antibody profiles against 4 SARS-CoV-2 antigens: the trimeric (whole) S-2P native spike protein, its RBD, its NTD (24), and N protein; the trimeric SARS-CoV spike protein; and spike proteins from circulating β-coronaviruses (HKU1, OC43) and α-coronaviruses (229E, NL63) , plus BSA, a negative control. Briefly, after blocking wells with 5% BSA, sera were added at 4 dilutions (1:100, 1:800, 1:3200, and 1:10,000) and incubated with shaking for 2 hours. Sulfo-tag–labeled anti-IgG detection antibodies were added, and the electrochemiluminescence signal was read using the MSD Sector 600 instrument (Meso Scale Diagnostics). Initial AUC of the electrochemiluminescence values for antibody detection were well above the BSA background for all sera for SARS-CoV-2 antigens, except for 1 sera for the RBD and N antigens and 10 sera for the NTD antigen. Samples were rescreened again in a second set of experiments after Meso Scale Diagnostics provided standards. Results are presented as dilution-corrected interpolated values from a standard curve with assigned AU/mL. Assignment of AU/mL of serum was performed by Meso Scale Diagnostics and is designed such that values are comparable with an International Standard Serum (ISS), so that bridging to a WHO International Standard will be possible in the future.

### Competition experiments.

Eight 2-fold dilutions of sera prediluted in a ratio of 1:50 assay diluent were added to an equal volume of assay diluent (control) or to assay diluent mixes containing 5 μg/mL SARS-CoV-2 spike and 5 μg/mL RBD proteins (SARS-CoV-2 RBD-spike cocktail), or 5 μg/mL spike proteins from all 4 circulating coronaviruses (HKU1, OC43, 229E, NL63; circulating coronaviruses [cCoVs] cocktail) (25), for an on-plate assay dilution of 1:100 through 1:12,800. The dilution series was incubated for 30 minutes at room temperature and then analyzed using the multiplex antibody assay protocol, as mentioned previously.

### CLIA antibody assay.

Total antibody (IgA, IgG, and IgM) against recombinant spike (S1) protein was determined using the VITROS 5600 analyzer (Ortho-Clinical Diagnostics) according to manufacturer instructions. This is a Health Canada and FDA-licensed qualitative assay with reported performance and in-house validation indicating sensitivities > 7 days after onset range between 96% and 100%, and specificities from 99% to 100% (26, 27).

### SPOT peptide array.

Forty-one 15-mer peptides selected based on their reactivity on convalescent samples and immunogenicity, and that were distributed over the entire SARS-CoV-2 proteome, were synthesized on a cellulose trioxatridecanediamine membrane using a MultiPep synthesizer (CEM) (28). Additionally, each membrane contained a human-IgG binding peptide as positive control (29). These in-house–made membranes were incubated with a 1:400 dilution of sera and incubated for 2 hours at room temperature. A copy of the array was also incubated with Tris buffered saline with Tween 20 only, as a negative control. After washing, membranes were incubated with secondary antibody (HRP-conjugated goat anti–human IgA + IgG + IgM polyclonal antibody, Jackson ImmunoResearch Inc., catalog 109-035-064) at a 1:30,000 dilution for another 2 hours, and detection was carried out using enhanced chemiluminescence detection, with 8 images captured over an exposure time of 50 seconds. The grayscale of images represented as numeric values between 0 and 10 were used before applying a uniform background correction of 1.

### Variables.

The following information was collected from participants by questionnaire: age; sex; the first 3 digits of their postal code; HCW status (and whether they worked at C&W or SMH); history of travel outside BC since January 1, 2020; and history of COVID-19 symptoms and testing. SARS-CoV-2–exposed cases were defined by a positive result on the commercial CLIA assay, validated for sensitivity by antibody profiling on the multiplex assay.

### Statistics.

The seroprevalence from a SARS-CoV-2 exposure was adjusted for bias due to false-positive and false-negative tests using the Greenland method (30). Differences in proportions were calculated using a Fisher’s exact test, with significance threshold at *P* < 0.05. Hierarchical clustering of antibody levels (based on the multiplex assay) was performed on log-transformed, *Z* score–normalized serology data, using the complete linkage agglomeration method and Euclidean distance measures. Spearman correlations between antibody levels and metavariables were adjusted for multiple testing using the Benjamini-Hochberg FDR = 0.05. There were no missing data. For competition experiments, same-sample groups were compared using 2-tailed paired *t* tests. Analyses were conducted in R version 4.0.2, R Studio version 3.6.2, and GraphPad Prism version 8.4.

### Study approval.

Written informed consent was obtained from all participants. The study procedures were approved by the University of British Columbia (UBC) C&W Research Ethic Board (H20-01205; H18-01724).

## Author contributions

AM, CM, and SD coordinated the study sample accrual and blood processing in Vancouver. JG and DM coordinated recruitment at SMH. CC collated the data and helped with data analysis. SEO performed the multiplex assay, with help from SN and MB. MG provided important input into the study design. JM and DE expressed and provided the soluble proteins for the multiplex assays and competition experiments. ACL, IS, and ANJ revised the manuscript. MI supervised the statistical analyses. VEB and DMG supervised the commercial CLIA testing of samples. DFHW and SP performed the SPOT peptide array analysis. PML and ABM supervised the study in Vancouver and at the NAID/NIH, respectively. AM, CM, SD, DCD, and PML wrote the manuscript’s first draft. All authors contributed to the study design, data analysis, and reviewing the manuscript, and they accept the article submission in its final form. The order of co–first authors was determined based on their earlier involvement at the study design stage.

## Supplementary Material

Supplemental data

## Version 1 03/15/2021

In-Press Preview

## Version 2 04/22/2021

Electronic publication

## Version 3 12/09/2021

In the Introduction, a year was corrected in the statement: "2.1 million deaths worldwide as of January 24, 2021"
