# Supplementary material for: A majority of uninfected adults show preexisting antibody reactivity against SARS-CoV-2
Source: JCI Insight. 2021 Apr 22;6(8):e146316. doi: 10.1172/jci.insight.146316 (PMC8119195; doi:10.1172/jci.insight.146316)
Supplement: Supplemental data [file jciinsight-6-146316-s005.pdf]

Supplemental material

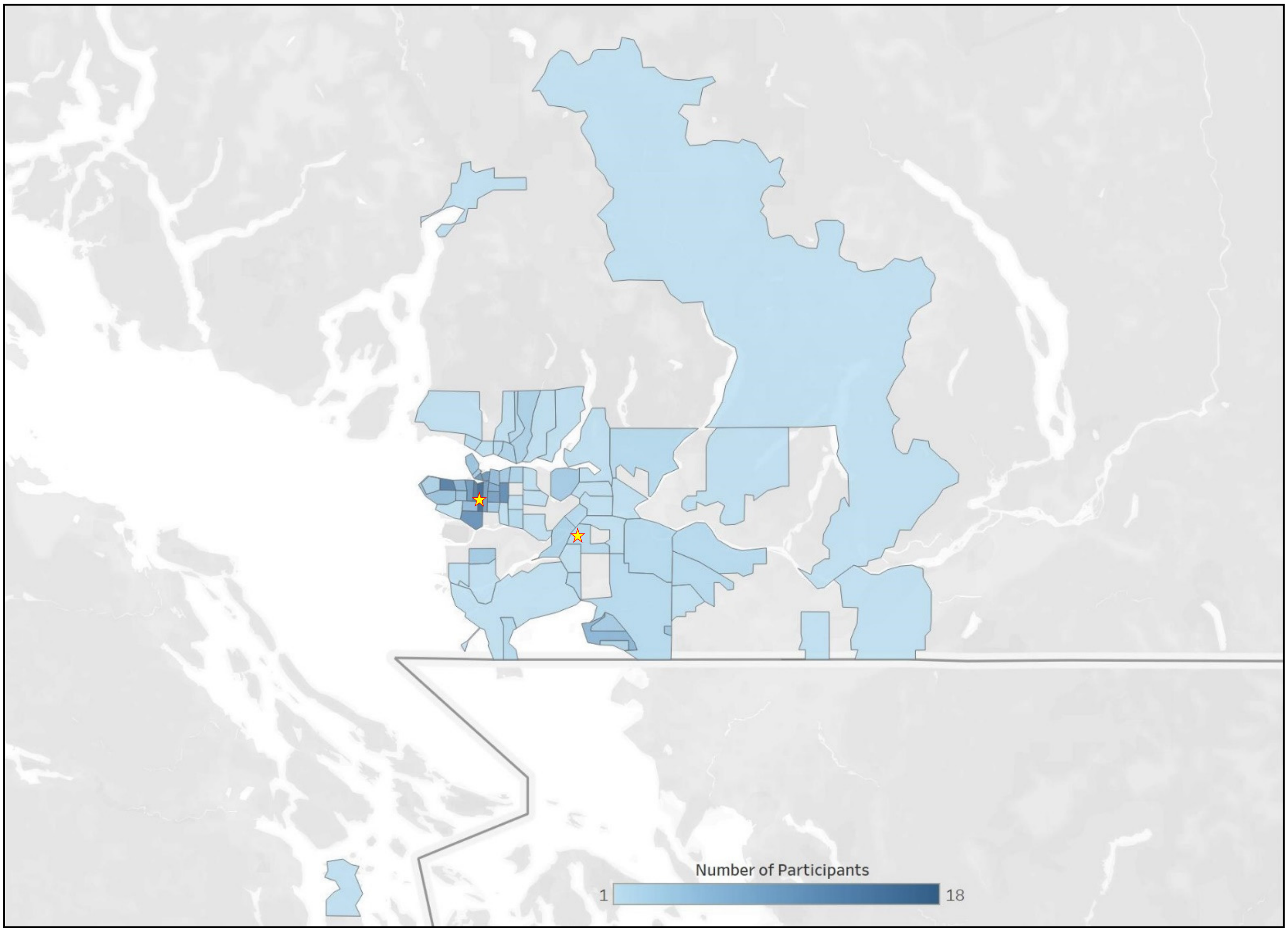

Supplemental Figure 1. Geographical distribution. Data are from 276 study participants, based on postal code information. Stars indicate the location of the two recruitment sites (BC Children's Hospital Research Institute on the left and Surrey Memorial Hospital on the right).

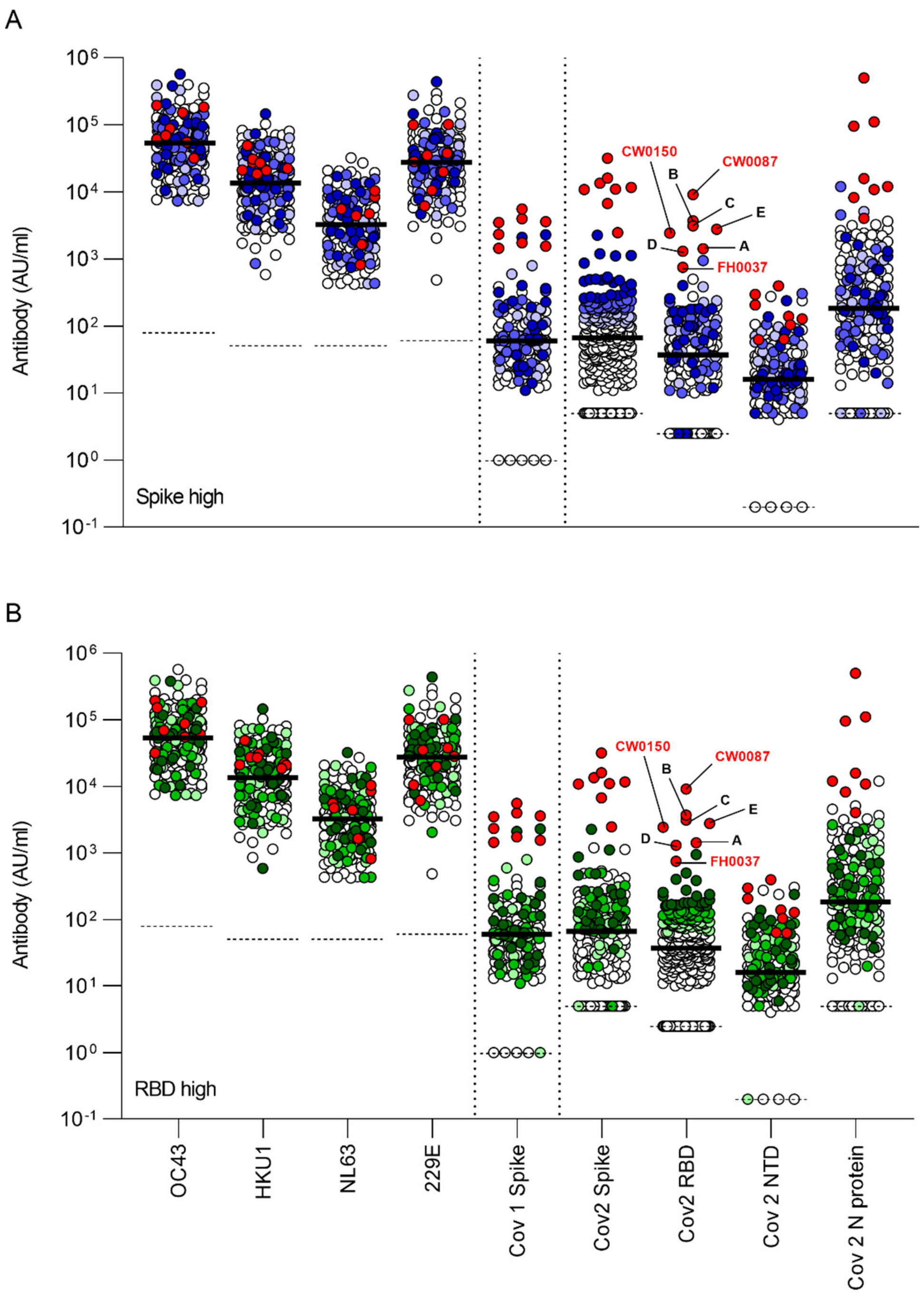

Supplemental Figure 2. Antibody reactivity to circulating coronavirus and SARS antigens on multiplex assay in 276 individuals in study cohort, plus 5 additional control convalescent sera. Data show antibody reactivity in SARS-CoV-2-infected individuals (in red), or in the 273 uninfected participants showing antibody reactivity against (**A**) spike or (**B**) RBD at the top 90th (darker tone), 80th (mid-tone) or 70th (lighter tone) centiles compared to the rest of the population. Thick black line = median. Thin dashed line = lower detection limit. Null values are shown at the detection limit.

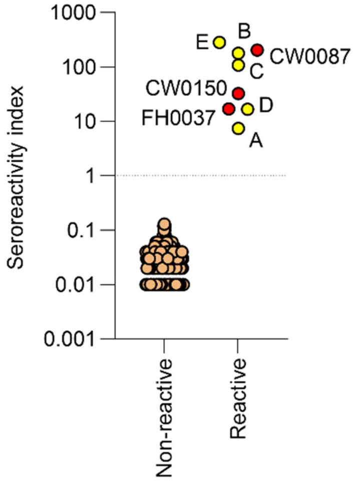

Supplemental Figure 3. Seroreactivity on the commercial CLIA assay among 222 of 273 unexposed individuals who tested above the mean for at least one of the four SARS-CoV-2 antigens in the multiplex ECLIA assay, identifies three reactive sera (red) and 219 non-reactive sera (orange) in the study cohort, based on the manufacturer's defined positive and negative thresholds of ≥1.00 and <1.00 (dotted line), respectively. Data also shows reactivity in the five additional convalescent sera (yellow), for comparison.

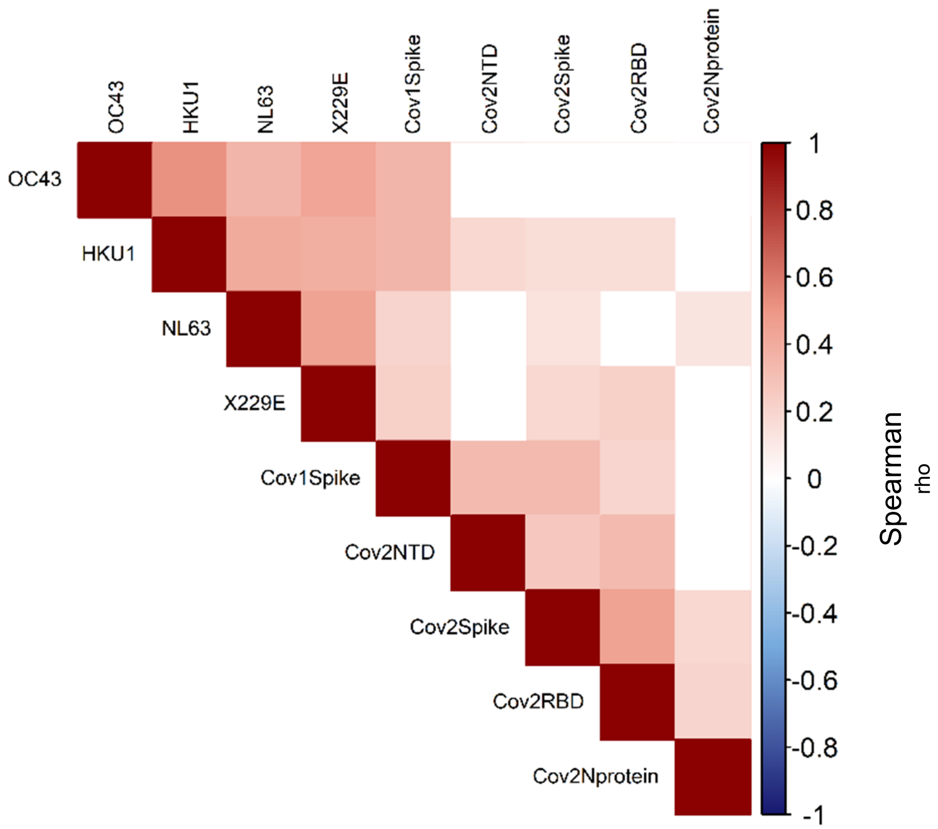

Supplemental Figure 4. Correlations among SARS-CoV-2 and circulating coronaviruses antibody levels. Spearman correlation was performed on serology data of the 273 seronegative individuals. Significant correlations are shown on colour scale, and ranged between 0.130 and 0.224 for 7 significant correlations with circulating coronaviruses (out of 36 correlations tested, FDR 5%; *P* and Spearman rho values are also provided in **Supplemental Table 3**).

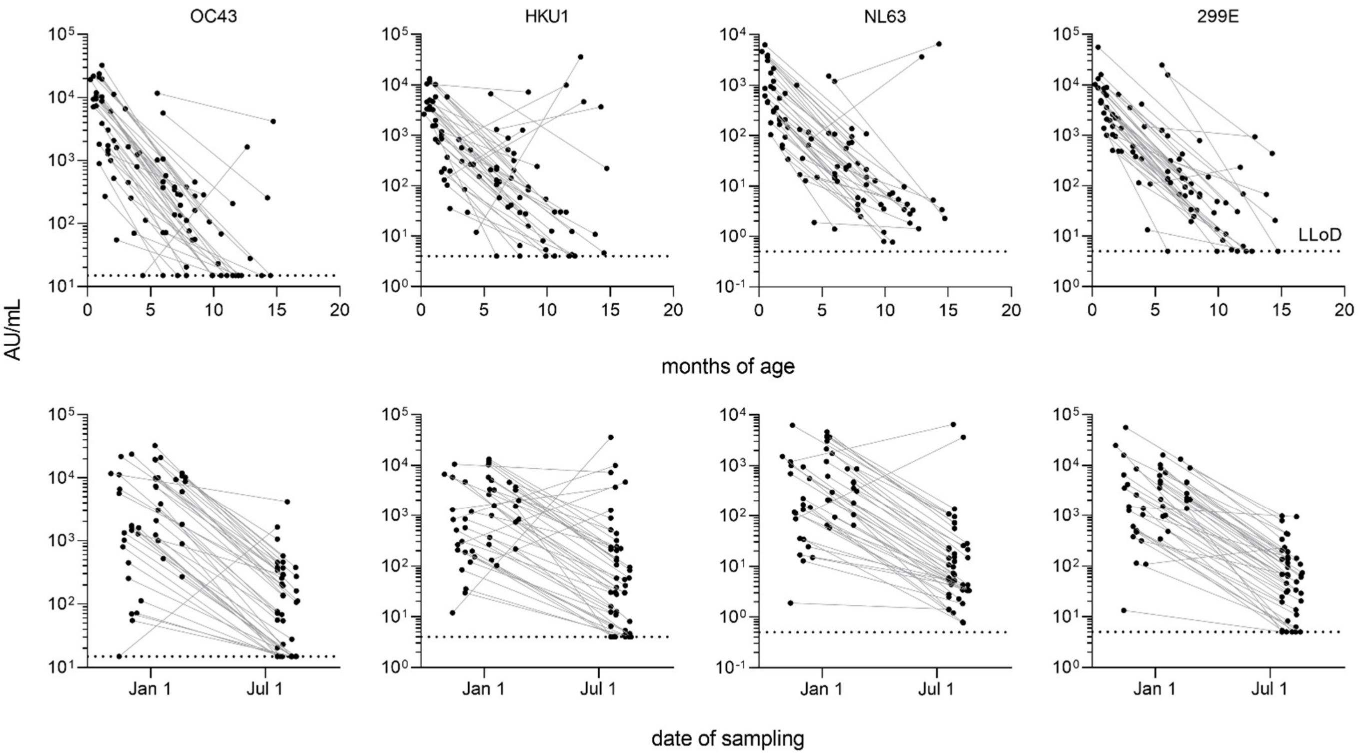

Supplemental Figure 5: Paired antibody reactivity for circulating coronaviruses in infants sampled before 6 months of age and again, ~8 months later (n = 45), by post-natal age (months) or date of sampling (2020); dotted line represents lower limit of detection (LLoD).

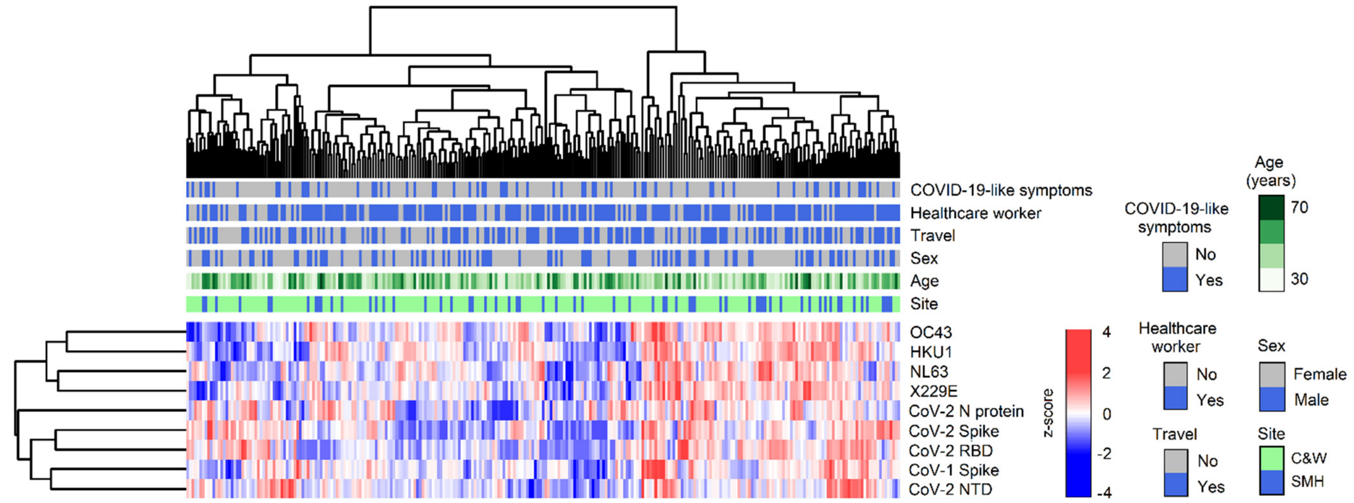

Supplemental Figure 6. Hierarchical clustering based on antibody levels against four SARS-CoV-2, SARS-1 spike antigens and human circulating coronaviruses from the 273 unexposed study participants by distribution of sociodemographic data. Colour scale represents antibody reactivity as a z-score.

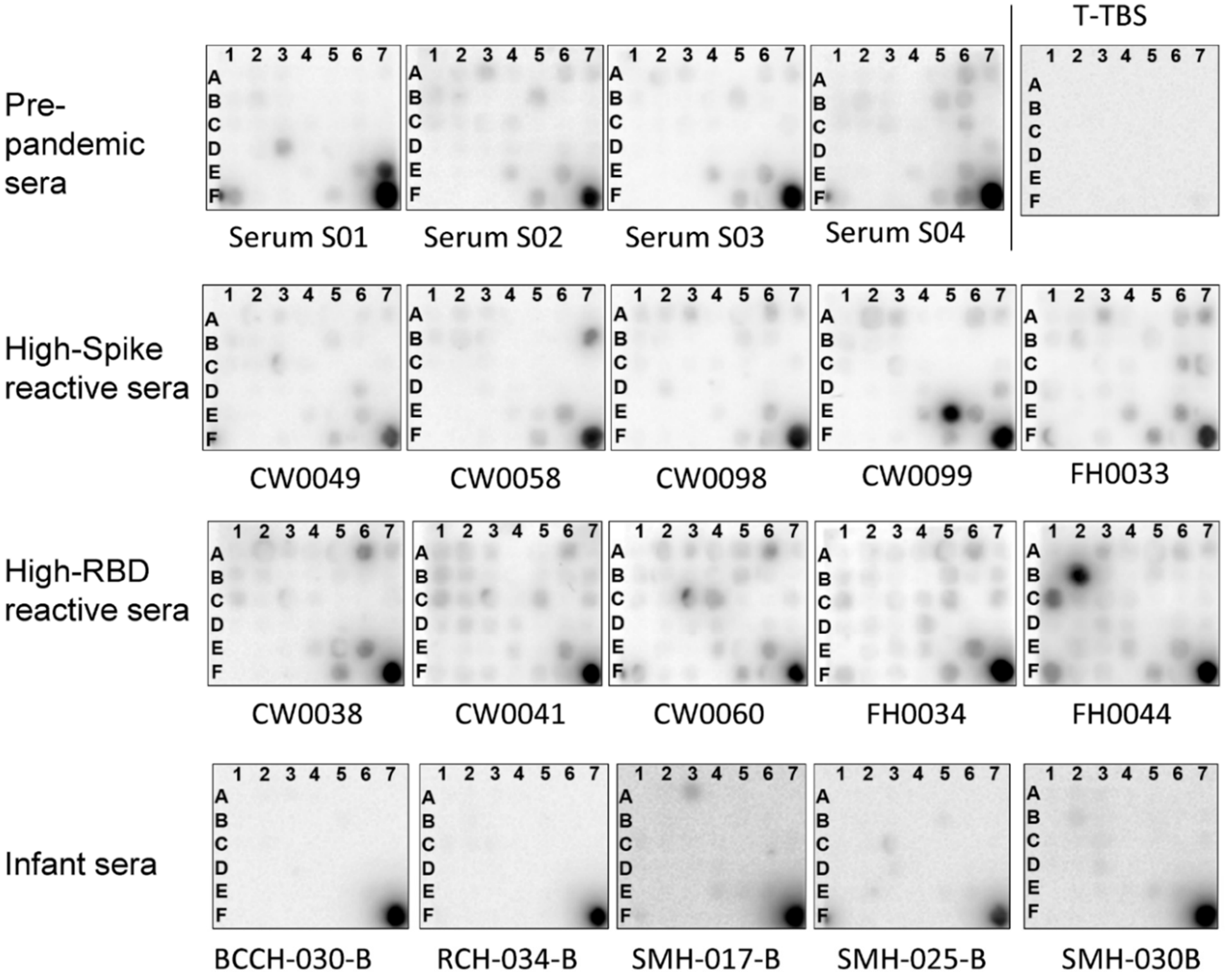

Array Position	Protein and Domain	Amino Acid Residue Span
A 1	Spike S1-NTD	40-54
A 2		44-58
A 3		174-188
A 4		252-266
A 5	Spike S1-RBD	315-329
A 6		322-336
A 7	Spike S1-RBM	463-477
B 1		491-505
B 2	Spike S1-CTD1	551-565
B 3		557-571
B 4		571-585
B 5	Spike S1-CTD2	626-641
B 6	Spike S2	713-727
B 7		765-779

Array Position	Protein and Domain	Amino Acid Residue Span
C 1	Spike S2	791-805
C 2		795-809
C 3	Spike S2-FP	813-827
C 4	Spike S2	879-893
C 5	Spike S2-HR1	925-939
C 6	S2-HR1/CH	975-989
C 7	S2-CH	987-1001
D 1		989-1003
D 2	S2-CD	1143-1157
D 3		1147-1161
D 4	Nucleocapsid N	13-27
D 5		156-170
D 6		241-255
D 7		271-285

Array Position	Protein and Domain	Amino Acid Residue Span
E 1	Nucleocapsid N	389-403
E 2	Membrane M	3-17
	Membrane M	7-21
E 6	ORF1a nsp2	153-167
	ORF1a nsp2	736-750
E 5	ORF1a nsp3	876-890
E 7	ORF1b nsp8/9	4133-4147
F 1	ORF1b nsp13	5447-5461
	ORF1b nsp13	5605-5619
F 3	ORF1b nsp14	6053-6067
F 4	ORF1b nsp15	6690-6704
	ORF1b nsp15	6722-6736
F 6	ORF3a	31-45
F 7	IgG Binding Peptide	N/A

Supplemental Figure 7. Representative SPOT peptide array membranes mapping SARS-Cov-2 antibody reactivity in uninfected individuals. Each spot represents represents antibody binding to a distinct 15-mer peptide. Tables represent the position of each peptide in the array the name of the protein or the domain as well as the animo acid span of the peptides. NTD: N-terminal and CTD: N- and C-terminal (Spike) domains; RBD: receptor-binding domain, HR: hinge region (between Spike S1 and S2 domains); HR: heptad repeat; CH: central helix; CD: connector domain; N: nucleocapsid; M: membrane protein; ORF: Open-Reading Frame polypeptide proteins; nsp: non-structural proteins.

Supplemental Table 1. Demographical characteristics of study participants.

**Participants' characteristics**	Total n = 276
Age, years, mean (standard deviation)	42.4 (11.9)
Sex, % female / male (n)	67.4 (186) / 32.6 (90)
Health worker occupation[£], % (n)	71.0% (196)
Travelled outside BC[¶], % (n)	50.3% (139)
Prior positive testing for COVID-19 (PCR), % (n)	0.72% (2)
COVID-19-associated symptoms, % (n)	25.0% (69)

BC: British Columbia, history of travel since January 1, 2020; [£]includes physicians; nurses; respiratory therapists; dieticians, genetic counsellors; psychologists; social workers; administrators; physiotherapists; occupational therapists; pharmacists; and pathologists. Complete data were available for all participants.

Supplemental Table 2. Clinical characteristics of three seroreactive study participants, plus five convalescent control sera.

Participant ID#	Age	Sex	Travel outside BC	Convalescence period (days)*	**Reported COVID-19-associated symptoms** (by open-ended question)
CW0087[£]	39	Female	No	57	Nasal congestion, nasal dripping, laryngitis, intermittent dry cough, fatigue, chills, anosmia/ageusia with gradual return over 3 months
CW0150[£]	24	Female	No	unknown[¶]	Asymptomatic
FH0037[£]	68	Male	Yes	72	Shortness of breath on exertion, generalized aching, fever, mild cough
A[€]	30	Female	No	75	Cough, rhinorrhea, generalized body ache, extreme fatigue, head congestion, fever, anosmia/ageusia
B[€]	35	Male	Yes	100	Fatigue, severe headache/body aches, shortness of breath, drenching night sweats, mild diarrhoea, very mild dry cough, pain in both feet persisting
C[€]	51	Male	Yes	96	Protracted cough, fatigue, mild coryza and conjunctivitis, head congestion, and mild sore throat
D[€]	28	Male	No	104	Mild shortness of breath on exertion
E[€]	25	Male	No	158	Sore throat, headache, rhinorrhea, head congestion, muscle aches, severe fatigue, anosmia/ageusia (persisting >6 months), orthostatic syncope

[£]Seroreactive study participants; [€]COVID-19 convalescent sera; *time between COVID-19 diagnosis by PCR and serology testing.
[¶]Retrospectively (after participant was made aware of positive serology testing) we were able to identify that she had a contact with a COVID-19 case 89 days prior to serology testing.

Supplemental Table 3. Spearman rho and false discovery rate-adjusted p values (FDR 5%) for correlations for antibody levels between SARS-CoV-2 and circulating coronaviruses.

	OC43	HKU1	NL63	X229E	Cov-1 spike	Cov-2 NTD	Cov-2 spike	Cov-2 RBD	Cov-2 N protein
OC43	0	3.60E-18	6.28E-09	6.04E-13	6.28E-09	0.062626	0.211963	0.364879	0.596884
	1	0.512986	0.355693	0.432947	0.355877	0.120348	0.078814	0.056223	0.032149
HKU1		0	2.28E-11	2.51E-10	6.28E-09	0.003351	0.014455	0.012611	0.083366
		1	0.405455	0.385529	0.355032	0.188397	0.157294	0.160958	0.111909
NL63			0	1.35E-13	0.001065	0.246839	0.026441	0.211963	0.045531
			1	0.444881	0.209192	0.072401	0.143556	0.078796	0.130119
X229E				0	0.000261	0.108618	0.003569	0.000441	0.087371
				1	0.233069	0.101703	0.185536	0.224499	0.109685
Cov-1 spike					0	7.49E-08	1.25E-07	0.001866	0.06245
					1	0.331146	0.324344	0.199227	0.121377
Cov-2 NTD						0	1.82E-05	1.00E-07	0.092581
						1	0.268994	0.327365	0.107155
Cov-2 spike							0	1.35E-13	0.003544
							1	0.446363	0.186518
Cov-2 RBD								0	0.001065
								1	0.209634
Cov-2 N protein									0
									1

FDR-adjusted p values are indicated in **black**
Spearman rho coefficients are indicated in **red**
